# Whole-genome sequencing identifies complex contributions to genetic risk by variants in genes causing monogenic systemic lupus erythematosus

**DOI:** 10.1007/s00439-018-01966-7

**Published:** 2019-02-01

**Authors:** Jonas Carlsson Almlöf, Sara Nystedt, Dag Leonard, Maija-Leena Eloranta, Giorgia Grosso, Christopher Sjöwall, Anders A. Bengtsson, Andreas Jönsen, Iva Gunnarsson, Elisabet Svenungsson, Lars Rönnblom, Johanna K. Sandling, Ann-Christine Syvänen

**Affiliations:** 1 Department of Medical Sciences, Molecular Medicine and Science for Life Laboratory, Uppsala University, 751 23 Uppsala, Sweden; 2 Division of Neuro and Inflammation Sciences, Department of Clinical and Experimental Medicine, Rheumatology, Linköping University, 581 83 Linköping, Sweden; 3 Department of Clinical Sciences, Rheumatology, Lund University, Skåne University Hospital, 222 42 Lund, Sweden; 4 Rheumatology Unit, Department of Medicine, Karolinska Institutet, Rheumatology, Karolinska University Hospital, 171 77 Stockholm, Sweden; 5 Department of Medical Sciences, Rheumatology and Science for Life Laboratory, Uppsala University, 751 85 Uppsala, Sweden

## Abstract

Systemic lupus erythematosus (SLE, OMIM 152700) is a systemic autoimmune disease with a complex etiology. The mode of inheritance of the genetic risk beyond familial SLE cases is currently unknown. Additionally, the contribution of heterozygous variants in genes known to cause monogenic SLE is not fully understood. Whole-genome sequencing of DNA samples from 71 Swedish patients with SLE and their healthy biological parents was performed to investigate the general genetic risk of SLE using known SLE GWAS risk loci identified using the ImmunoChip, variants in genes associated to monogenic SLE, and the mode of inheritance of SLE risk alleles in these families. A random forest model for predicting genetic risk for SLE showed that the SLE risk variants were mainly inherited from one of the parents. In the 71 patients, we detected a significant enrichment of ultra-rare ( ≤ 0.1%) missense and nonsense mutations in 22 genes known to cause monogenic forms of SLE. We identified one previously reported homozygous nonsense mutation in the *C1QC* (Complement C1q C Chain) gene, which explains the immunodeficiency and severe SLE phenotype of that patient. We also identified seven ultra-rare, coding heterozygous variants in five genes (*C1S, DNASE1L3, DNASE1, IFIH1*, and *RNASEH2A*) involved in monogenic SLE. Our findings indicate a complex contribution to the overall genetic risk of SLE by rare variants in genes associated with monogenic forms of SLE. The rare variants were inherited from the other parent than the one who passed on the more common risk variants leading to an increased genetic burden for SLE in the child. Higher frequency SLE risk variants are mostly passed from one of the parents to the offspring affected with SLE. In contrast, the other parent, in seven cases, contributed heterozygous rare variants in genes associated with monogenic forms of SLE, suggesting a larger impact of rare variants in SLE than hitherto reported.

**Electronic supplementary material:**

The online version of this article (10.1007/s00439-018-01966-7) contains supplementary material, which is available to authorized users.

## Introduction

Systemic lupus erythematosus (SLE, OMIM 152700) is a clinically heterogeneous autoimmune disease with an estimated heritability of 0.66 similar to other autoimmune diseases (Selmi et al. 2012). In the past decade, genome-wide association studies (GWAS) have identified more than 100 risk loci that are robustly associated with SLE (Chen et al. 2017; Langefeld et al. 2017). The risk variants identified by GWAS are rarely located in protein-coding exons, instead most of them are common variants thought to affect regulatory genomic regions such as promoters and enhancers (Hindorff et al. 2009; Farh et al. 2015).

In addition, there exist several monogenic disorders with an SLE-like phenotype that are inherited in a Mendelian fashion and are caused by mutations in one out of 32 so far known genes (Tsokos et al. 2016). These genes have been identified by familial manifestation of SLE that is mainly shared between mother and daughter or between female sibling pairs in a family. In ten of these genes there are mutations that cause classical SLE where a patient fulfills the classification criteria for SLE (Tan et al. 1982). Another set of 12 genes carry mutations that cause dysregulation of genes in the type I interferon (IFN) system, which is a prominent feature shared by the majority of patients with SLE (Hagberg and Ronnblom 2015).

In aggregate, monogenic forms of SLE contribute only to a small fraction of all SLE cases. The most common form of monogenic SLE is caused by mutations in the *TREX1* gene that have been identified in 0.5–2% of adult SLE patients (Lee-Kirsch et al. 2007; Namjou et al. 2011). The highest penetrance of an SLE-like disease has been observed for mutations in the complement system, with a particularly high penetrance for complement factor 1 and 4 deficiencies, while a lower penetrance has been observed for the more common complement factor 2 deficiency (Pickering et al. 2000). The variants in complement system genes represent less than 1% of all SLE cases combined. Highly penetrant monogenic diseases manifest when a protein-coding gene is affected by mutations in one or both alleles, depending on if the deleterious allele is recessive or dominant. A recessive disease-causing effect can be the result of a homozygous deleterious genetic variant or by compound heterozygosity in a protein-coding gene where different deleterious variants have been inherited from each parent. However, more subtle effects of heterozygous mutations have been observed for variants connected to Mendelian diseases (Sidransky 2006; Valente and Ferraris 2007) blurring the line between Mendelian and complex disorders.

To increase the power of finding associations for rare mutations in a case–control association setting, there are a number of tests that combine the effect of several variants within a region of interest into one test. Examples of these are burden tests (Morgenthaler and Thilly 2007; Han and Pan 2010) and variance component tests (Wu et al. 2011). An even broader approach is to test for enrichment of variants in selected features in a set of genes (Singh et al. 2017). A completely global approach is to use machine learning on all called variants to be able to separate healthy individuals from patients (Abraham and Inouye 2015). We have previously used this approach in SLE where we trained a random forest model using the variants from 1160 patients and 2711 controls genotyped on the ImmunoChip to obtain a SLE risk score (Almlof et al. 2017).

Using whole-genome sequencing (WGS) of parent-offspring trios, it is possible to find almost all single nucleotide variants (SNVs) and most smaller insertions–deletions (INDELs), while at the same time identifying the parent of origin for many of the variants. Whole exome sequencing (WES) of SLE family trios has identified *de novo* mutations and potential novel SLE genes (Pullabhatla et al. 2018). WES has also successfully identified rare variants that are likely pathogenic in SLE (Delgado-Vega et al. 2018) and WGS of monozygotic twins discordant for SLE has found CNVs that may be associated with difference in SLE phenotype between twins (Chen et al. 2018).

In this study, we performed whole-genome sequencing (WGS) of samples from 71 Swedish SLE trio families with two healthy parents and one child affected by SLE. We employed the trio study design to investigate rare risk variants for SLE located in functional elements in, and in the vicinity of, genes carrying variants that are known to cause monogenic disorders with an SLE-like phenotype. Using a combination of WGS trio data with the previously trained random forest, it was possible to investigate the parent of origin for called variants and elucidate possible differences in inheritance depending on sex and type of variants.

## Results

### Risk of SLE from common SNPs is mainly inherited from one parent

In an earlier study (Almlof et al. 2017), we developed a random forest (RF) model to determine a score that indicates the risk to develop SLE based on the genotype data from a Swedish SLE case–control association study using the ImmunoChip with approximately 120 k SNPs across 186 loci known to be associated with immune-mediated diseases (Illumina) (Cortes and Brown 2011). We here used the single nucleotide variant (SNV) calls from WGS of 71 trio families with the offspring affected by SLE that overlap with the SNVs included on the ImmunoChip (97.4% overlap) to determine the RF derived risk scores for SLE for the trio family members. We used the scores to compare the risk of SLE for the parents in the trio families with that of healthy Swedish controls (*n* = 2711) and to compare the risk scores for the patients with SLE in the trio families with the risk scores for the larger cohort of SLE patients, who were included in the ImmunoChip case–control study (*n* = 1160). According to the prediction by the RF model, the parents in the trio families had a higher average SLE disease score than the healthy controls (34% vs 27%), but a lower average disease score than the SLE patients in the trio families (34% vs 42%). The average risk of SLE for the parent with the higher risk of SLE in each family was of similar magnitude as that for the patients (42%), while the parent with lower risk of SLE displayed an equally low risk of SLE as the controls (26%). These risk predictions indicate that the complex genetic predisposition for SLE is mainly inherited to the patient from one of the parents in a family.

**Fig. 1:**
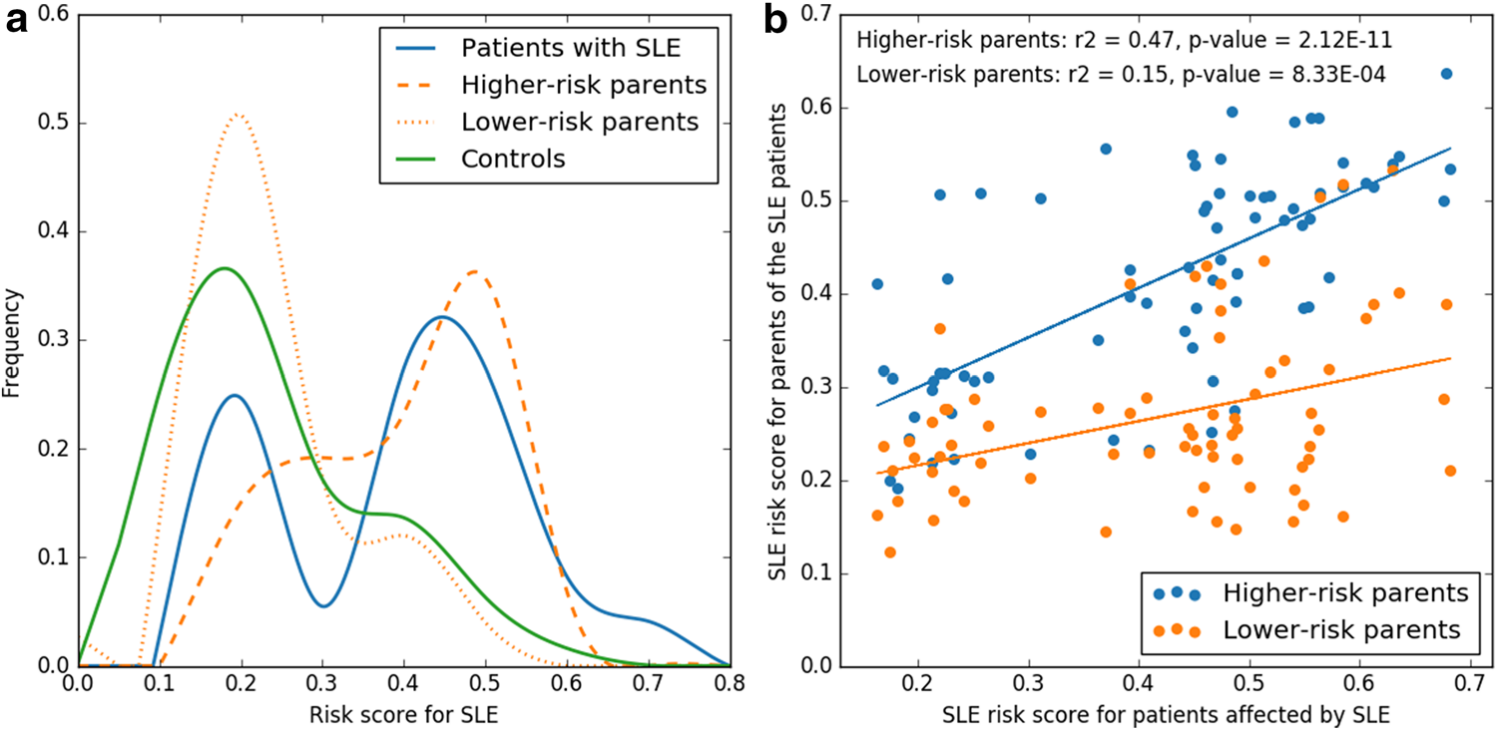
Risk score for systemic lupus erythematosus (SLE) of parents and patients with SLE in the family trios. **a** Distribution of predicted random forest risk scores for SLE patients (*n* = 71), their parents and healthy controls. The two parents in each family are separated into higher and lower risk based on their respective random forest risk score. **b** Linear correlation between the random forest risk score for SLE of the patients and of the parent with higher SLE risk score in each family trio is shown in blue. The correlation between the SLE risk score for the SLE patient and the parent with lower risk of SLE in each family is shown in orange

Support for the one-parent mode of inheritance is provided in (Fig. 1a) where the distribution of the risk scores for SLE between the members of the trio families show similarities between the parents with the higher SLE risk score and the SLE patients, while the distribution for the parents with a lower SLE risk score show similar distribution as the controls. Another way to illustrate this is through correlation of the risk score of the SLE patients and of the parents (Fig. 1b). There is a highly significant correlation coefficient of 0.47 (*p* value 2.12E − 11) between the risk scores for the parent with the higher risk of SLE in each family and those of the SLE patients in the trios. The correlation coefficient of 0.47 should be compared to that of the parents with a lower risk score of SLE, who had a correlation coefficient of only 0.15 with the SLE risk of the patients in the trio, where the correlation is mainly driven by a few high-risk samples. Notably, there was no difference in average risk scores between the mothers and the fathers.

### Enrichment of ultra-rare missense variants in genes associated with monogenic SLE

Next, we investigated if the variants called in WGS data from our patients with SLE were enriched in promoter and protein-coding regions of SLE genes in comparison to the recently published Swedish genomes reference dataset [SweGen (Ameur et al. 2017)]. For the variants in protein-coding regions, we only considered non-silent variants. The enrichment analysis included variants in 22 genes that are reported to cause monogenic forms of classical SLE or dysregulation of the type I interferon system (Supplemental Table S1).

**Fig. 2:**
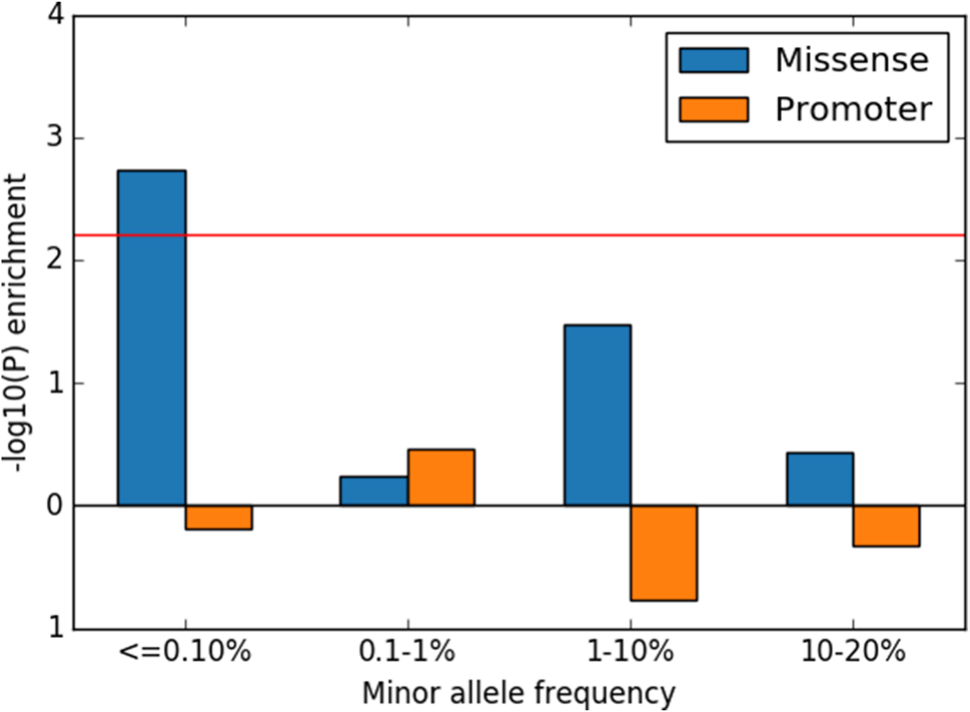
Enrichment analysis of missense and promoter variants. *p* values for enrichment of missense and promoter variants in genes causing monogenic forms of SLE are shown on the vertical axis at different minor allele frequencies as indicated on the horizontal axis. The red line shows the 0.05 significance threshold after multiple testing correction. Values below zero on the horizontal axis indicate depletion

In the SLE patients from the trio families, we observed an enrichment (OR = 2.07, *p* value = 0.00182) of ultra-rare missense variants with minor allele frequency (MAF) ≤ 0.1% in protein-coding regions of genes known to cause monogenic forms of SLE (Fig. 2). The majority (20 out of 21) of these ultra-rare sequence variants was observed in the heterozygous form. The 21 ultra-rare sequence variants identified in 18 patients represent an excess of 10.9 variants compared to that expected by chance according to the enrichment analysis. Thus, approximately one-seventh of the SLE patients included in our analysis seem to carry rare risk variants with small to medium effect sizes in one of the genes causing monogenic SLE. For variants with higher MAF and variants in promoters, we did not observe any significant enrichment. Variants close to genes that have previously been associated to SLE in GWAS studies were also investigated in a similar fashion as the genes associated with monogenic SLE but no significant enrichment was found.

### Functional annotation of rare variants in genes causing monogenic SLE

The potential functional impact in SLE of each of the 21 rare SLE risk variants was assessed based on their functional annotations, effects or locations in the encoded proteins, DANN score, and predicted effect on the protein function by the SIFT or PolyPhen2 programs. In one of the patients, we found a previously reported homozygous nonsense mutation in the *C1QC* gene (Arg69*) (Schejbel et al. 2011). A non-functional C1q protein leads to lupus-like symptoms with 85% penetrance and to SLE that fulfills the American College of Rheumatology (ACR) criteria for classification of SLE (Tan et al. 1982) with 50% penetrance (van Schaarenburg et al. 2016). The patient with the homozygous nonsense mutation in the *C1QC* gene suffers from immunodeficiency and a severe SLE phenotype (Bolin K, Eloranta M-L, Kozyrev SV, Dahlqvist J, Nilsson B, Knight A, Rönnblom L, manuscript in preparation). In addition, we detected seven heterozygous missense or truncating mutations in seven patients located in five genes (*C1S, DNASE1L3, DNASE1, IFIH1*, and *RNASEH2A*) with high potential to contribute to SLE. The identified variants are described in detail in Table 1 and calling quality measures for the variants are listed in Supplemental Table S2, showing the high reliability of the variant calling. Two of the genes (*DNASE1* and *IFIH1)* contain two unique mutations. Five of the variants are reported in dbSNP, all with low MAF in Europeans and at most 0.05% MAF in the SweGen reference dataset (Ameur et al. 2017). However, two of the variants found in *DNASE1* have a markedly higher MAF in African populations. The last two variants are not found at all in the Swedish reference population or in dbSNP. Each of the variants was only found in one patient.

**Table 1:** Summary of missense and nonsense variants that are predicted to affect function identified in 71 trio families in genes carrying known variants causing monogenic SLE

Gene Amino acid change Nucleotide change	Protein function	DANN^a^ SIFT^b^ PolyPhen2^c^	Effect of mutation	RF risk score of patient with variant
*C1S* P09871-1:p.(Asp631Asn) NC_000012.11:g.7177779G > A	Complement C1s subcomponent (C1s) that together with C1q and C1r forms C1, which is the first component in the classical pathway of the complement system. C1s activates C2 and C4 by cleaving the protein chain at specific sites (Venkatraman Girija et al. 2013)	0.999 0.0 (D) 1.0 (D)	The mutation is located next to one of the active site residues responsible for protein C2 and C4 cleavage and is therefore likely to reduce the catalytic activity of the enzyme	0.68
*C1QC* P02747-1:p.(Arg69*) NC_000001.10.g:22973743C > T	C-chain polypeptide of serum complement subcomponent C1q, which associates with C1r and C1s to yield the first component of the serum complement system	NA NA NA	Nonsense mutation giving rise to a non-functional C1q protein (Schejbel et al. 2011), which leads to lupus-like symptoms with 85% penetrance and to SLE with 50% penetrance (van Schaarenburg et al. 2016)	0.25
*DNASE1* P24855-1:p.(Gly127Arg) NC_000016.9:g.3706697G > A rs8176919	Deoxyribonuclease-1 cleaves DNA during apoptosis and necrosis (Errami et al. 2013). Together with deoxyribonuclease gamma (coded by *DNASE1L3)*, it is one key component in degradation of neutrophil extracellular traps (Jimenez-Alcazar et al. 2017)	0.999 0.035 (D) 1.0 (D)	Present in Africans (AFR) at 7% MAF, but are virtually non-existent in European populations. The Gly127Arg mutation is located in the sharp hair-pin bend of a loop coordinating one of two Ca2 + ions required for its catalytic activity (Parsiegla et al. 2012)	0.26
*DNASE1* P24855-1:p.(Pro154Ala) NC_000016.9:g.3707023C > G rs1799891	Deoxyribonuclease-1 cleaves DNA during apoptosis and necrosis (Errami et al. 2013). Together with deoxyribonuclease gamma (coded by *DNASE1L3)*, it is one key component in degradation of neutrophil extracellular traps (Jimenez-Alcazar et al. 2017)	0.989 0.045 (D) 0.235 (B)	Found in Africans (AFR) at 2% MAF, but are virtually non-existent in European populations. The Pro154Ala is located only two amino acids from the active site at His156. The large change in amino acid properties could reduce the DNA cleaving efficiency of the protein	0.22
*DNASE1L3* Q13609-1:p.(Thr224Met) NC_000003.11:g.58183581G > A	*DNASE1L3* encodes the protein deoxyribonuclease gamma that cleaves DNA during apoptosis and necrosis (Errami et al. 2013). Together with deoxyribonuclease-1 (coded by *DNASE1)*, it is a key component in degradation of neutrophil extracellular traps (Jimenez-Alcazar et al. 2017)	0.999 0.026 (D) 1.0 (D)	Based on the homologous structure of DNASE1 (PDB ID: 3W3D), the Thr224Met mutation affects an amino acid in a loop where the surrounding residues (223, 225–230) coordinate Ca2 + binding, which is critical for the activity of the protein (Yakovlev et al. 2000)	0.55
*IFIH1* Q9BYX4-1:p.(Arg77Trp) NC_000002.11:g.163174589G > A rs147278787	Interferon-induced helicase C domain-containing protein 1 induces type I interferons and proinflammatory cytokines upon viral infection (Gitlin et al. 2006)	0.999 0.002 (D) 0.998 (D)	The Arg77Trp mutation is located in the first of the two CARD domains of IFIH1, which interacts with the CARD domains of other proteins to induce antiviral signaling (Wu et al. 2013). The large change in amino acid property and close to maximal DANN score suggest that the mutations affect interactions of the CARD domain	0.58
*IFIH1* Q9BYX4-1:p.(Arg374Cys) NC_000002.11:g.163139062G > A rs113854430	Interferon-induced helicase C domain-containing protein 1 induces type I interferons and proinflammatory cytokines upon viral infection (Gitlin et al. 2006)	0.998 0.078 (T) 0.993 (D)	The Arg374Cys mutation is structurally close to the mutations Arg337Gly, Leu372Phe, Arg720Gln, and, Arg779His that have been shown to either enhance the IFNB1 promoter activation or enhance activation of the interferon pathway in addition to causing the SLE-like disease AGS	0.18
*RNASEH2A* O75792-1:p.(Lys221Arg) NC_000019.9:g.12923921A > G rs143534021	Encodes the catalytic subunit of RNase HII called ribonuclease H2 subunit A that removes unwanted ribonucleotides from DNA. Defective removal of ribonucleotides from DNA has been shown to promote systemic autoimmunity in a dose response manner (Gunther et al. 2015)	0.362 0.699 (T) 0.001 (B)	The Lys221Arg mutation is structurally close to variants causing SLE-like disease AGS (Thr240Met, Arg245Gly, Phe230Leu). The mutation is reported in Clinvar as being of uncertain significance regarding AGS. It introduces only small changes in amino acid properties. But small reduction of the activity of this enzyme could increase the risk of SLE (Gunther et al. 2015)	0.47

*D* damaging or deleterious, *T* tolerated, *B* benign, *AGS* Aicardi–Goutières syndrome (OMIM: 610333, 615846)

^a^The DANN score ranges from 0 to 1, where 1 represents the highest possibility for pathogenicity

^b^Predicted *p* value of the variant being damaging

^c^Predicted probability of the variant being deleterious

### Mode of inheritance of rare risk variants

**Fig. 3:**
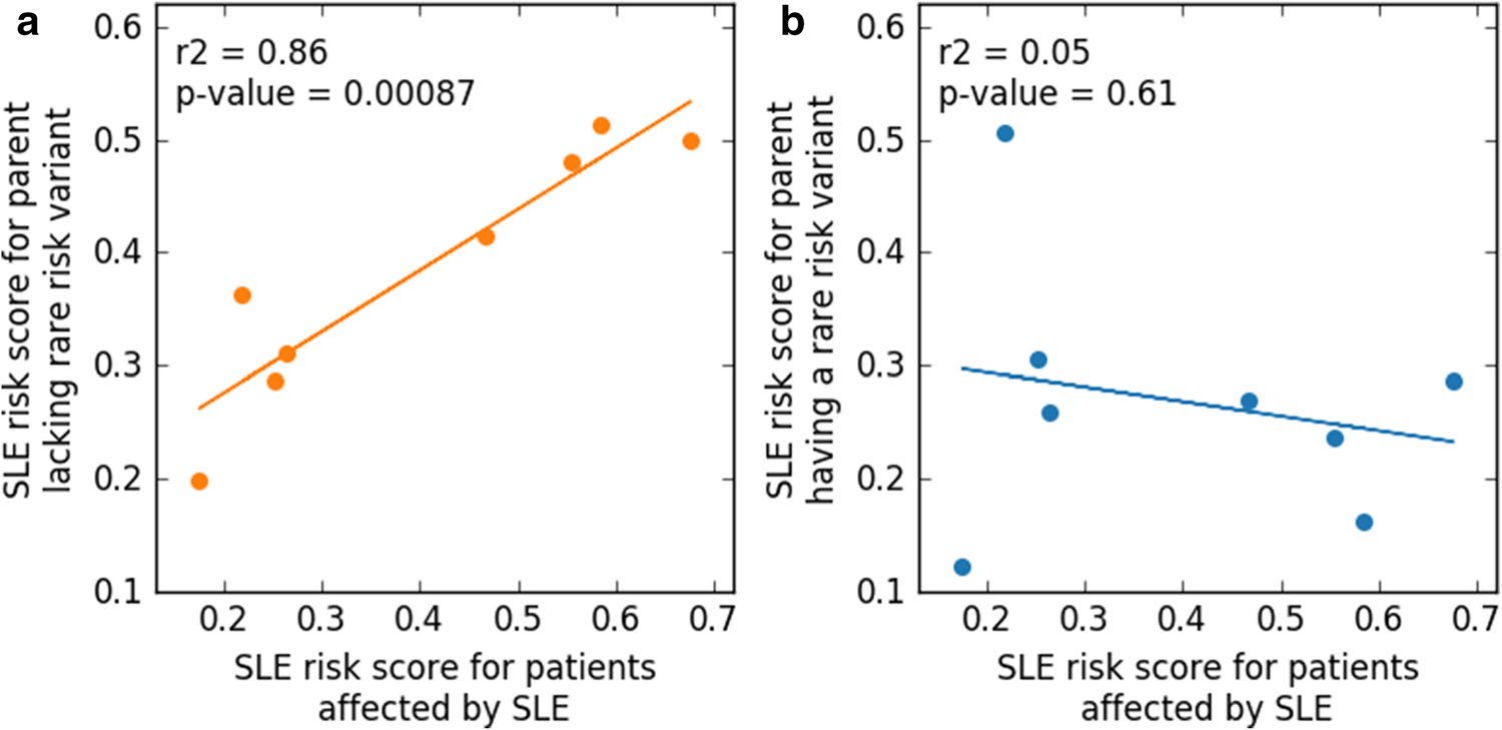
Linear correlation between the random forest risk score for SLE of the patients and the parents with and without any of the eight reported rare variants. **a** The orange line shows the high correlation between the RF risk score for the parent lacking the rare variant and the patient. **b** The blue line shows the absence of correlation between the parent carrying the rare variant and the SLE patient

To examine the mode of inheritance of the eight rare risk variants for SLE reported in Table 1, we investigated if there were any patterns that showed from which of the parents the variant was inherited or if it was randomly inherited. The SLE risk scores for the eight patients with the rare risk variant were not significantly different from the other patients in the study. However, the inheritance of the risk score was not randomly distributed. We found that there was a high correlation (*R*^2^ = 0.86) between the RF risk score of the parent lacking the SLE risk variant identified in Table 1 and the patient (Fig. 3a). On the other hand, no correlation was observed between the RF risk score of the parent having the SLE risk variant and the RF risk score of the patient (Fig. 3b). Thus, the genetic burden of SLE in the child is mostly inherited from one of the parents with the added burden from the other parent in the form of the rare risk variant identified here.

### Clinical characteristics of patients with heterozygous rare risk variants

By comparing the frequencies of SLE sub-phenotypes, as described by the 11 ACR criteria, between the seven patients with heterozygous rare risk variants with all patients in this study, we were able to distinguish if this sub-group presented a unique disease manifestation. Strikingly, none of the patients with heterozygous rare risk variants had nephritis compared to 38% in the entire cohort. However, the difference are only nominally significant (*p* = 0.022) before multiple testing correction for the 11 ACR criteria tested. None of the other ACR criteria show any trends between the patient groups.

## Discussion

Rare genetic variants that have remained undetected due to limitations in statistical power are believed to be one of the causes of the “missing heritability” observed despite many large GWAS of complex diseases. Burden or aggregate association tests, in which all rare variants affecting the same gene are combined into one test, are used to increase the statistical power for rare variant association. Some recent studies have succeeded in identifying genes with rare variants with statistical significance, exemplified by *RNASEH2* in SLE (Gunther et al. 2015), whilst rare variants in other genes have failed to be replicated, like *SIAE* in RA (Surolia et al. 2010; Hunt et al. 2011). Here, to further increase the statistical power, we simultaneously analyzed rare variants in multiple genes that have been shown to cause Mendelian forms of SLE. Using this approach, it is not possible to observe association between individual genes and SLE, instead we obtain a measure of the enrichment of disease-contributing rare variants in all tested genes. However, we are limited in power by the low number of samples studied. We will therefore only pick up the strongest signals and might miss weaker signals present in for example promoters, enhancers, or variants at different minor allele frequencies. In addition, reproducibility of the exact reported variants is problematic due to the rarity of the variants. On the other hand, the enrichment of rare variants in genes associated to monogenic SLE should be easier to confirm.

The enrichment of SNVs in the genes causing monogenic SLE was calculated by comparison with the reference genomes of a thousand healthy individuals that constitute the SweGen dataset (Ameur et al. 2017). The variant calling procedure differs between our study and the SweGen dataset as we utilize the trio information to improve the variant calling accuracy. This will have the greatest impact on private variants as they will gain support from at least one parent in our study. To minimize this effect, we normalized the enrichment based on the difference in the total number of variant calls between the two datasets in the relevant minor allele frequency range and annotated functional elements.

As shown in Fig. 1a, the distribution of the risk scores generated using a random forest model for SLE patients is bimodal. This could partly be a consequence of the low sample size. However, a less pronounced bimodal distribution of the risk scores remains when including all the 1160 genotyped patients to construct the random forest predictor, which suggests that a bimodal distribution is an accurate representation of the data, and that two distinct groups of patients with differing genetic risk for SLE exist within the SLE patient population studied here. However, there is no significant association between risk scores and any of the ACR criteria, sex, or age of onset. The apparent difference in SLE risk is instead probably mainly due to the fact that the ImmunoChip does not cover all the variations found in SLE. The ImmunoChip targets only approximately 120 k SNPs across 186 loci known to be associated with immune-mediated diseases and thus most of the rare variations will remain undetected.

In our study, we found that the RF risk scores of the parents without the rare risk variant had a high correlation with the RF risk score of the patient (Fig. 3a). The parents with the rare risk variant on the other hand showed no such correlation (Fig. 3b). This observation suggests that the risk variants with higher minor allele frequencies are inherited from one parent and that the additional genetic burden needed to trigger SLE in the child is inherited from the other parent in the form of a very rare risk variant affecting a gene known to cause monogenic SLE. To draw a parallel to cancer, it would constitute the second hit needed to develop the disease. These patients could also be viewed as a new subgroup of SLE patients with an intermediate genetic risk compared to the patients with monogenic SLE and those with high-frequency risk variants found by GWAS.

Most of the ultra-rare candidate risk variants for SLE identified in our study encode amino acids located close to functionally critical amino acid residues, but they may not be critical alone. For example, variants in *C1S* and *DNASE1* are located close to active sites of these enzymes, variants in *DNASE1* and *DNASE1L3* affect the Ca^2+^ binding loop in the corresponding proteins, but are not involved in the actual binding, variants in *IFIH1* and *RNASEH2A* are spatially close to known SLE-like disease-causing variants in the proteins. Such variants could affect the protein function, but it seems unlikely that they could cause complete inactivation of the protein, instead they might contribute to increased risk for SLE in a similar fashion as common risk variants identified by GWAS. Two of the genes (*DNASE1* and *IFIH1)* carry two unique mutations providing extra functional support for these. In addition, the two rare variants in *DNASE1* have markedly higher minor allele frequencies in African populations than in Europeans, which could possibly explain part of the 3–4 times higher prevalence of SLE in African populations (McCarty et al. 1995).

The random forest model calculates a SLE risk score which when compared with the risk of healthy individuals can be used to the probability to develop SLE. However, as the disease is rare, even a greatly increased risk would still equal a quite low probability to develop SLE in a single individual, implying that the random forest model in its present form would not be useful in a clinical setting.

## Materials and methods

### DNA samples

DNA was extracted from peripheral whole blood of 71 SLE patients and their biological parents attending the rheumatology clinics of the university hospitals in Uppsala, Stockholm (Karolinska University Hospital), Lund, and Linköping (Supplemental Table S3). All patients were examined by a rheumatologist and the medical records were reviewed. SLE patients and their parents provided informed consent to participate in the study, and the study was approved by the regional ethics committees. Of the patients 85% were female and averaged 24 years old at SLE onset. The patients fulfilled at least four American College of Rheumatology (ACR) 1982 criteria for SLE (Tan et al. 1982), with the exception of five patients who displayed three ACR criteria together with a clinical diagnosis of SLE, see further Supplemental Table S4. None of the parents had SLE at the time of sample collection and the average age of the parents was over 50 years of age.

### Whole-genome sequencing and sequence alignment

Sequencing libraries were prepared from 1 µg of DNA using reagents from the TruSeq PCR-free DNA sample preparation kit (Illumina Inc.) targeting an insert size of 350 bp. 150 bp paired-end whole-genome sequencing was performed on an Illumina HiSeqX sequencer using v2.5 sequencing chemistry (Illumina Inc.). Whole-genome sequencing (WGS) was performed by the SNP&SEQ Technology Platform at Uppsala University, Sweden (http://www.sequencing.se). The sequences were aligned with BWA (Li and Durbin 2009) version 0.7.12 using default parameters and the b37 human reference from the GATK file bundle version 2.8. The reads in the raw alignments were then flagged for duplication and recalibrated using GATK version 3.3.0 (McKenna et al. 2010). The number of average aligned reads was 920 million per sample, which corresponds to an average genomic coverage of 40X. Statistics of the WGS after mapping and variant calling are shown in Supplemental Table S5.

### Calling single nucleotide variants (SNVs)

Variants in the WGS data were called jointly in all samples using GATK version 3.5.0 following the GATK best practice protocol (Van der Auwera et al. 2013). In the variant recalibration step, we used positive training data from Hapmap (phred quality score prior likelihood of Q15 which is equal to 97% likelihood that the genotype is correct) and 1000 Genomes Omni 2.5M chip (prior Q12, 94% likelihood) as well as in-house genotype data from the same samples from the Infinium OmniExpressExome-8 v1.3 SNP chip (Illumina) with 958497 SNP markers (prior Q20, 99% likelihood). As additional training data, we used the 1000 Genomes high confidence calls (prior Q10, 90% likelihood) and for annotation and statistics the dbSNP version 138 (prior Q2, 37% likelihood). All data files except the in-house SNP genotype data were obtained from the GATK file bundle version 2.8. Variants were marked as PASS if the variant quality score log-odds (VQSLOD) were higher than the 99th percentile in the training data for SNVs. The variants were then further refined by calculating genotype posterior using the data from parent-offspring trios in GATK. Low quality variants were flagged if the genotype posterior had a score < Q20.

### Gene enrichment analysis

Enrichment analysis was performed for 22 genes (supplemental Table S1) known to be involved in monogenic forms of SLE (Tsokos et al. 2016). The analyzed genes cause either monogenic SLE fulfilling four ACR criteria (10 genes) or a SLE-like disease by affecting the type I interferon pathway (12 genes). The odds ratios and enrichment were calculated in relation to the background frequencies in the SweGen reference dataset containing 1000 whole-genome sequenced Swedish individuals sequenced to similar depth and at the same sequencing facility as our data (Ameur et al. 2017). The enrichment analysis was performed for variants affecting the coding sequence and for variants in promoter. The data were then normalized based on the ratio for all variants in the relevant annotations and allele frequencies between the two studies.

### Annotation of SNVs

The variants from all datasets were annotated using Annovar version 2016.05.11 (Wang et al. 2010). Chromatin state annotations of promoters were obtained from the ChromHMM (Ernst and Kellis 2012) predictions for the B-lymphocyte cell line GM12878. Relative gene positions were obtained from the RefSeq database (Pruitt et al. 2007). Minor allele frequencies in the Swedish population were retrieved from the SweGen database (Ameur et al. 2017) and from the European samples in the 1000 Genomes project (Genomes Project et al. 2015). Known SNVs were annotated using dbSNP release 138 (Sherry et al. 2001). The effect of nsSNVs on the encoded proteins was according to the predictions by SIFT (Kumar et al. 2009) and PolyPhen2 (Adzhubei et al. 2010). For identifying potential pathogenic variants, the DANN score (Quang et al. 2015) was used, where 1.0 is maximal pathogenic potential and 0.0 is minimal potential. The DANN score together with the Combined Annotation-Dependent Depletion (CADD) score have the best performance to discriminate germline pathogenic mutations according to recent benchmarks (Drubay et al. 2018).

## Conclusion

We found that the higher minor allele frequency risk variants for SLE are mainly inherited to the patient from one of the parents in a trio family, while in some cases the second parent contributes with rare risk variants in genes causing monogenic forms of SLE. Based on enrichment analysis in functional elements, 11 of the 21 risk variants identified in our study should contribute to SLE, while we found evidence for eight of the identified variants to have an effect on the function of the encoded protein. Thus, rare variants in genes known to cause monogenic SLE could contribute to the risk of SLE in one out of nine patients which suggests a larger impact of rare variants in SLE than hitherto reported. In the absence of a replication cohort and functional validation of the rare variants reported here, future studies are needed to confirm these findings.

## Electronic supplementary material

Below is the link to the electronic supplementary material.

Supplementary material 1 Clinical information of all the patients in the study (XLSX 16 KB)

Supplementary material 2 (DOCX 23 KB)

## Data Availability

Rare variants presented in this paper have been submitted to dbSNP (https://www.ncbi.nlm.nih.gov/projects/SNP/).

## References

[CR1] Abraham G, Inouye M (2015). Genomic risk prediction of complex human disease and its clinical application. Curr Opin Genet Dev.

[CR2] Adzhubei IA, Schmidt S, Peshkin L (2010). A method and server for predicting damaging missense mutations. Nat Methods.

[CR3] Almlof JC, Alexsson A, Imgenberg-Kreuz J (2017). Novel risk genes for systemic lupus erythematosus predicted by random forest classification. Sci Rep.

[CR4] Ameur A, Dahlberg J, Olason P (2017). SweGen: a whole-genome data resource of genetic variability in a cross-section of the Swedish population. Eur J Hum Genet.

[CR5] Chen L, Morris DL, Vyse TJ (2017). Genetic advances in systemic lupus erythematosus: an update. Curr Opin Rheumatol.

[CR6] Chen F, Li Z, Li R, Li Y (2018). Wholegenome sequencing of a monozygotic twin discordant for systemic lupus erythematosus. Mol Med Rep.

[CR7] Cortes A, Brown MA (2011). Promise and pitfalls of the Immunochip. Arthritis Res Ther.

[CR8] Delgado-Vega AM, Martinez-Bueno M, Oparina NY (2018). Whole exome sequencing of patients from multicase families with systemic lupus erythematosus identifies multiple rare. Var Sci Rep.

[CR9] Drubay D, Gautheret D, Michiels S (2018). A benchmark study of scoring methods for non-coding mutations. Bioinformatics.

[CR10] Ernst J, Kellis M (2012). ChromHMM: automating chromatin-state discovery and characterization. Nat Methods.

[CR11] Errami Y, Naura AS, Kim H (2013). Apoptotic DNA fragmentation may be a cooperative activity between caspase-activated deoxyribonuclease and the poly(ADP-ribose) polymerase-regulated DNAS1L3, an endoplasmic reticulum-localized endonuclease that translocates to the nucleus during apoptosis. J Biol Chem.

[CR12] Farh KK, Marson A, Zhu J (2015). Genetic and epigenetic fine mapping of causal autoimmune disease variants. Nature.

[CR13] Genomes Project C, Auton A, Brooks LD (2015). A global reference for human genetic variation. Nature.

[CR14] Gitlin L, Barchet W, Gilfillan S (2006). Essential role of mda-5 in type I IFN responses to polyriboinosinic:polyribocytidylic acid and encephalomyocarditis picornavirus. Proc Natl Acad Sci USA.

[CR15] Gunther C, Kind B, Reijns MA (2015). Defective removal of ribonucleotides from DNA promotes systemic autoimmunity. J Clin Investig.

[CR16] Hagberg N, Ronnblom L (2015). Systemic lupus erythematosus—a disease with a dysregulated type I interferon system Scandinavian. J Immunol.

[CR17] Han F, Pan W (2010). A data-adaptive sum test for disease association with multiple common or rare variants. Hum Hered.

[CR18] Hindorff LA, Sethupathy P, Junkins HA, Ramos EM, Mehta JP, Collins FS, Manolio TA (2009). Potential etiologic and functional implications of genome-wide association loci for human diseases and traits. Proc Natl Acad Sci USA.

[CR19] Hunt KA, Smyth DJ, Balschun T (2011). Rare and functional SIAE variants are not associated with autoimmune disease risk in up to 66,924 individuals of European ancestry. Nat Genet.

[CR20] Jimenez-Alcazar M, Rangaswamy C, Panda R (2017). Host DNases prevent vascular occlusion by neutrophil extracellular traps. Science.

[CR21] Kumar P, Henikoff S, Ng PC (2009). Predicting the effects of coding non-synonymous variants on protein function using the SIFT algorithm. Nat Protoc.

[CR22] Langefeld CD, Ainsworth HC, Cunninghame Graham DS (2017). Transancestral mapping and genetic load in systemic lupus erythematosus. Nat Commun.

[CR23] Lee-Kirsch MA, Gong M, Chowdhury D (2007). Mutations in the gene encoding the 3′-5′ DNA exonuclease TREX1 are associated with systemic lupus erythematosus. Nat Genet.

[CR24] Li H, Durbin R (2009). Fast and accurate short read alignment with Burrows-Wheeler transform. Bioinformatics.

[CR25] McCarty DJ, Manzi S, Medsger TA, Ramsey-Goldman R, LaPorte RE, Kwoh CK (1995). Incidence of systemic lupus erythematosus race gender differences. Arthritis Rheum.

[CR26] McKenna A, Hanna M, Banks E (2010). The genome analysis toolkit: a MapReduce framework for analyzing next-generation DNA sequencing data. Genome Res.

[CR27] Morgenthaler S, Thilly WG (2007). A strategy to discover genes that carry multi-allelic or mono-allelic risk for common diseases: a cohort allelic sums test (CAST). Mutat Res.

[CR28] Namjou B, Kothari PH, Kelly JA (2011). Evaluation of the TREX1 gene in a large multi-ancestral lupus cohort. Genes Immun.

[CR29] Parsiegla G, Noguere C, Santell L, Lazarus RA, Bourne Y (2012). The structure of human DNase I bound to magnesium and phosphate ions points to a catalytic mechanism common to members of the DNase I-like superfamily. Biochemistry.

[CR30] Pickering MC, Botto M, Taylor PR, Lachmann PJ, Walport MJ (2000). Systemic lupus erythematosus, complement deficiency, and apoptosis. Adv Immunol.

[CR31] Pruitt KD, Tatusova T, Maglott DR (2007). NCBI reference sequences (RefSeq): a curated non-redundant sequence database of genomes, transcripts and proteins. Nucleic Acids Res.

[CR32] Pullabhatla V, Roberts AL, Lewis MJ (2018). De novo mutations implicate novel genes in systemic lupus erythematosus. Hum Mol Genet.

[CR33] Quang D, Chen Y, Xie X (2015). DANN: a deep learning approach for annotating the pathogenicity of genetic variants. Bioinformatics.

[CR34] Schejbel L, Skattum L, Hagelberg S (2011). Molecular basis of hereditary C1q deficiency–revisited: identification of several novel disease-causing mutations. Genes Immun.

[CR35] Selmi C, Lu Q, Humble MC (2012). Heritability versus the role of the environment in autoimmunity. J Autoimmun.

[CR36] Sherry ST, Ward MH, Kholodov M, Baker J, Phan L, Smigielski EM, Sirotkin K (2001). dbSNP: the NCBI database of genetic variation. Nucleic Acids Res.

[CR37] Sidransky E (2006). Heterozygosity for a Mendelian disorder as a risk factor for complex disease. Clin Genet.

[CR38] Singh T, Walters JTR, Johnstone M (2017). The contribution of rare variants to risk of schizophrenia in individuals with and without intellectual disability. Nat Genet.

[CR39] Surolia I, Pirnie SP, Chellappa V (2010). Functionally defective germline variants of sialic acid acetylesterase in autoimmunity. Nature.

[CR40] Tan EM, Cohen AS, Fries JF (1982). The 1982 revised criteria for the classification of systemic lupus erythematosus. Arthritis Rheum.

[CR41] Tsokos GC, Lo MS, Costa Reis P, Sullivan KE (2016). New insights into the immunopathogenesis of systemic lupus erythematosus. Nat Rev Rheumatol.

[CR42] Valente EM, Ferraris A (2007). Heterozygous mutations in genes causing Parkinsonism: monogenic disorders go complex. Lancet Neurol.

[CR43] van Schaarenburg RA, Magro-Checa C, Bakker JA (2016). C1q deficiency and neuropsychiatric systemic lupus erythematosus. Front Immunol.

[CR44] Van der Auwera GA, Carneiro MO, Hartl C (2013). From FastQ data to high confidence variant calls: the genome analysis toolkit best practices pipeline. Curr Protoc Bioinform.

[CR45] Venkatraman Girija U, Gingras AR, Marshall JE (2013). Structural basis of the C1q/C1s interaction and its central role in assembly of the C1 complex of complement activation. Proc Natl Acad Sci USA.

[CR46] Wang K, Li M, Hakonarson H (2010). ANNOVAR: functional annotation of genetic variants from high-throughput sequencing data. Nucleic Acids Res.

[CR47] Wu MC, Lee S, Cai T, Li Y, Boehnke M, Lin X (2011). Rare-variant association testing for sequencing data with the sequence kernel association test. Am J Hum Genet.

[CR48] Wu B, Peisley A, Richards C (2013). Structural basis for dsRNA recognition, filament formation, and antiviral signal activation by MDA. 5 Cell.

[CR49] Yakovlev AG, Wang G, Stoica BA, Boulares HA, Spoonde AY, Yoshihara K, Smulson ME (2000). A role of the Ca^2+^/Mg^2+^-dependent endonuclease in apoptosis and its inhibition by poly(ADP-ribose) polymerase. J Biol Chem.

