# Supplementary material for: Whole-genome sequencing identifies complex contributions to genetic risk by variants in genes causing monogenic systemic lupus erythematosus
Source: Hum Genet. 2019 Feb 1;138(2):141–50. doi: 10.1007/s00439-018-01966-7 (PMC6373277; doi:10.1007/s00439-018-01966-7)
Supplement: Supplementary file 2 — Supplementary material 2 (DOCX 23 KB) [file 439_2018_1966_MOESM2_ESM.docx]

# Whole-genome sequencing identifies complex contributions to genetic risk by variants in genes causing monogenic systemic lupus erythematosus

## Human Genetics

Jonas Carlsson Almlöf^1*^, Sara Nystedt^1^, Dag Leonard^5^, Maija-Leena Eloranta^5^, Giorgia Grosso^4^, Christopher Sjöwall^2^, Anders A. Bengtsson^3^, Andreas Jönsen^3^, Iva Gunnarsson^4^, Elisabet Svenungsson^4^, Lars Rönnblom^5^, Johanna K. Sandling^5^, Ann-Christine Syvänen^1^

^1^Department of Medical Sciences, Molecular Medicine and Science for Life Laboratory, Uppsala University, 751 23 Uppsala, Sweden; ^2^Department of Clinical and Experimental Medicine, Rheumatology/Division of Neuro and Inflammation Sciences Linköping University, 581 83 Linköping, Sweden; ^3^Lund University, Skåne University Hospital, Department of Clinical Sciences, Rheumatology, 222 42 Lund, Sweden; ^4^Rheumatology Unit, Department of Medicine, Karolinska Institutet, Rheumatology, Karolinska University Hospital, 171 77 Stockholm, Sweden; ^5^Department of Medical Sciences, Rheumatology and Science for Life Laboratory, Uppsala University, 751 85 Uppsala, Sweden; *Correspondence; Tel: +4618-471 4680, Email: jonas.carlsson@medsci.uu.se

Supplemental Table S1. Summary of genes associated with monogenic SLE included in the enrichment analysis, type of SLE-like disease, and the affected pathway relevant for SLE.

| Gene | Disease | Pathway |
| --- | --- | --- |
| *ACP5* | Spondyloenchondrodysplasia with immune dysregulation | Type I IFN, nucleic acid sensing |
| *ADAR* | Aicardi–Goutières syndrome 6 | Type I IFN, nucleic acid sensing |
| *C1QA* | SLE, Complement C1 deficiency | Immune complex and waste clearance |
| *C1QB* | SLE, Complement C1 deficiency | Immune complex and waste clearance |
| *C1QC* | SLE, Complement C1 deficiency | Immune complex and waste clearance |
| *C1R* | SLE, Complement C1 deficiency | Immune complex and waste clearance |
| *C1S* | SLE, Complement C1 deficiency | Immune complex and waste clearance |
| *C4A* | SLE, Complement C1 deficiency | Immune complex and waste clearance |
| *C4B* | SLE, Complement C1 deficiency | Immune complex and waste clearance |
| *DDX58* | Singleton–Merten syndrome 2 | Type I IFN, nucleic acid sensing |
| *DNASE1* | SLE | Nucleic acid sensing |
| *DNASE1L3* | SLE | Nucleic acid sensing |
| *FASLG* | SLE, Autoimmune lymphoproliferative syndrome 1B | Lymphocyte signalling |
| *IFIH1* | Aicardi–Goutières syndrome 7 (AGS7) | Type I IFN, nucleic acid sensing |
| *ISG15* | Immunodeficiency 38, with basal ganglia calcification | Type I IFN, nucleic acid sensing |
| *PSMB8* | Nakajo syndrome | Type I IFN, immune complex and waste clearance |
| *RNASEH2A* | Aicardi–Goutières syndrome 4 (AGS4) | Type I IFN, nucleic acid sensing |
| *RNASEH2B* | Aicardi–Goutières syndrome 2 (AGS2) | Type I IFN, nucleic acid sensing |
| *RNASEH2C* | Aicardi–Goutières syndrome 3 (AGS3) | Type I IFN, nucleic acid sensing |
| *SAMHD1* | Aicardi–Goutières syndrome 5 (AGS5) | Type I IFN, nucleic acid sensing |
| *TMEM173* | STING-associated vasculopathy, infantile-onset | Type I IFN, nucleic acid sensing |
| *TREX1* | Aicardi–Goutières syndrome 1 (AGS1) | Type I IFN, nucleic acid sensing |

Supplemental Table S2. Variant calling quality metrics for the reported rare variants in Table 1.

| Chrom | Pos | QUAL | Average coverage | MQ^1^ | MQ0^2^ | VQSLOD^3^ | Parent / offspring | Ref. reads | Alt. allele reads |
| --- | --- | --- | --- | --- | --- | --- | --- | --- | --- |
| 1 | 22973743 | 1511.04 | 32.3 | 60 | 0 | 19.4 | Offspring | 0 | 18 |
|  |  |  |  |  |  |  | Parent | 9 | 23 |
|  |  |  |  |  |  |  | Parent | 15 | 13 |
| 2 | 163139062 | 1835.53 | 37.0 | 60 | 0 | 16.7 | Offspring | 22 | 16 |
|  |  |  |  |  |  |  | Parent | 36 | 33 |
| 2 | 163174589 | 526.26 | 32.3 | 60 | 0 | 17.7 | Offspring | 18 | 11 |
|  |  |  |  |  |  |  | Parent | 8 | 12 |
| 3 | 58183581 | 622.26 | 35.2 | 60 | 0 | 17.7 | Offspring | 16 | 15 |
|  |  |  |  |  |  |  | Parent | 22 | 13 |
| 12 | 7177779 | 773.26 | 35.3 | 60 | 0 | 16.8 | Offspring | 14 | 19 |
|  |  |  |  |  |  |  | Parent | 28 | 15 |
| 16 | 3706697 | 654.26 | 33.3 | 60 | 0 | 18.0 | Offspring | 22 | 14 |
|  |  |  |  |  |  |  | Parent | 13 | 13 |
| 16 | 3707023 | 1074.53 | 34.5 | 60 | 0 | 18.4 | Offspring | 21 | 17 |
|  |  |  |  |  |  |  | Parent | 10 | 11 |
| 19 | 12923921 | 552.26 | 35.0 | 60 | 0 | 17.4 | Offspring | 12 | 15 |
|  |  |  |  |  |  |  | Parent | 18 | 9 |

^1^MQ = mapping quality as determined by BWA-mem. Max value = 60. ^2^MQ0 = Number of reads with mapping quality = 0. ^3^VQSLOD = Variant quality score log-odds.

Supplemental Table S3. Distribution of trio families between regional hospitals in Sweden.

| **Center** | **Number of trios** | **Number of affected females** |
| --- | --- | --- |
| Uppsala | 22 | 19 |
| Linköping | 4 | 1 |
| Lund | 15 | 12 |
| Stockholm | 30 | 28 |
| **Total** | 71 | 60 (85%) |

Supplemental Table S5. Metrics from the whole-genome sequencing of samples from trio families.

| **Statistics** | **Average (Standard deviation)** |
| --- | --- |
| Coverage | 40.4 (7.4) |
| Coverage > 30X | 79.5% (11.3%) |
| Aligned reads | 920M (16M) |
| Percent aligned reads | 99.3% (0.5%) |
| Duplication rate | 12.7% (4.6%) |
| Insert size | 360 (20.4) |
| GC content | 40.8% (0.3%) |
| Ti/Tv ratio | 2.10 |
| SNVs^1^ | 13355113 |
| INDELs^1^ | 3309183 |
| Insertions^1^ | 772838 |
| Deletions^1^ | 998747 |
| Missense SNV^1^ | 191725 |
| Nonsense SNV^1^ | 2754 |
| Silent SNV^1^ | 159421 |
| Variants per sample^1^ | 3740689 |
| Homozygous variants per sample^1^ | 2265531 |
| Heterozygous variants per sample^1^ | 1475158 |

^1^For the variants, the counts include only variant that passed filters.
